# Validity of self-report measures of cannabis use compared to biological samples among women of reproductive age: a scoping review

**DOI:** 10.1186/s12884-022-04677-0

**Published:** 2022-04-21

**Authors:** Kara R. Skelton, Erin Donahue, Sara E. Benjamin-Neelon

**Affiliations:** 1 Department of Health Sciences, College of Health Professions, 251 Towson Way, Towson, MD 21204 USA; 2 Department of Occupational Therapy and Occupational Science, College of Health Professions, 251 Towson Way, Towson, MD 21204 USA; 3 Department of Health, Behavior and Society, Johns Hopkins Bloomberg School of Public Health, 615 North Wolfe St, Baltimore, MD 21205 USA

**Keywords:** Marijuana, Pregnancy, Perinatal, Substance use

## Abstract

**Background:**

Most existing evidence about the prevalence of prenatal cannabis use relies on self-reported measures, which is limited by social desirability bias and recall bias. To date, several studies have examined the validity of self-reported measures of prenatal cannabis use, but this evidence has yet to be synthesized. To address this gap, we performed a scoping review to systematically identify and synthesize existing evidence on the validity of self-reported measures of cannabis use among pregnant women.

**Methods:**

We searched PubMed, PyschINFO, CINAHL, Cochrane/CENTRAL, and Google Scholar for peer-reviewed studies published in English between January 2010 and June 2021. We included studies that compared self-reported measures of cannabis use to a biochemical measure of cannabis (e.g., urine, hair, meconium) in pregnant women. We excluded studies reporting solely on prenatal cannabis use prevalence as well as those that examined self-reported drug use in which cannabis use was not a distinct category.

**Results:**

We found 12 unique studies (11 primary studies and one systematic review) that examined the validity of self-reported prenatal cannabis use, compared to a biochemical sample. Most studies were conducted in the US and conducted in either a hospital or clinical setting. We found that self-report was more valid in populations with a current or prior history of drug use. Self-report was also more valid when assessed via interviews by research team members than health care provider screenings or self-administered surveys. The most commonly used biochemical measure used was urine drug testing, which was found to have the highest level of concordance with self-report.

**Conclusions:**

This scoping review systematically mapped existing evidence on the validity of self-reported prenatal cannabis use. Although much remains unknown in this area, an important next step is a systematic review that would provide robust evidence on clinical utilization of self-reported use in conjunction with biochemical samples. Further research is needed to examine validity by type of measure and mode of administration. Additionally, future studies could assess factors associated with disclosure of use across different critical maternal health periods beyond pregnancy.

**Supplementary Information:**

The online version contains supplementary material available at 10.1186/s12884-022-04677-0.

## Background

Substantial increases in cannabis use prevalence among women of reproductive age over the past decade is a global public health concern [1–4]. Of particular concern is rising use of cannabis during pregnancy – a critical period for both women and their offspring [5–7]. These stark upticks in prenatal cannabis use can be seen in North America, particularly the United States (US) and Canada [2, 8, 9]. In the US, estimates of prenatal cannabis use more than doubled from 3.4% in 2002–2003 to 7.0% in 2016–2017 [10]. Similarly, Canada saw a relative increase of 61% in prenatal cannabis use prevalence from 2012 to 2017 [2].

Emerging evidence indicates prenatal cannabis use and exposure is not without consequence. Indeed, a recent systematic review and meta-analysis by Marchand et al. found that in-utero cannabis exposure was associated with an increased risk of several adverse neonatal health outcomes compared with infants not exposed [11]. Neonates with in utero cannabis exposure had higher rates of preterm birth, low birth weight, small for gestational age, admission to the neonatal intensive care unit, and smaller head circumference [11]. Importantly, THC can persist in breast milk up to 6 weeks after prenatal cannabis use cessation [12], which has large implications for pregnant women who use cannabis and intend to breastfeed. A recent call to action highlighted the growing body of evidence supporting risk of adverse neonatal health outcomes associated with in utero cannabis exposure [13]. Collectively, prenatal care clinicians are integral to preventing these adverse health outcomes via enhanced screening for and clear communication about risks of cannabis use and exposure.

Effective screening for cannabis use during pregnancy is essential for prevention of adverse perinatal consequences associated with in-utero cannabis exposure. However, most studies reporting on prenatal cannabis use rely on maternal self-report [8–10, 14–16]. Additionally, these self-report measures are relatively quick and easy to administer in clinical settings [17]. Self-report also allows for patient contextual elaboration regarding prenatal cannabis use, including frequency and mode of administration. However, there are many limitations of self-reported measures, including stigma and fear of punitive consequences, especially in high-risk populations, such as pregnant women [18, 19]. Additionally, the setting, interviewer, and population have also been shown to influence the validity of self-report [20]. Thus, examining the validity of self-reported measures of prenatal cannabis use across diverse populations with different administrators in different settings is of clinical importance. Accurate detection of prenatal cannabis use is also an important methodological issue for studies that aim to examine the effect of cannabis use and exposure on perinatal health outcomes. To date, several studies have assessed the validity of self-reported measures of prenatal cannabis use in comparison to estimates from biochemical samples, such as urine, hair, umbilical cord, or meconium samples [17, 21–24]. However, this evidence has yet to be reviewed and synthesized.

To fill this evidence gap, we performed a scoping review that systematically identified and synthesized existing evidence on the validity of self-reported measures of prenatal cannabis use in comparison to estimates from biochemical samples. In conducting this scoping review, we also aimed to determine if there was enough available evidence on this topic to perform a future systematic review and identify potential questions that could be answered with existing evidence.

## Methods

We aligned this scoping review with the Preferred Reporting Items for Systematic Reviews and Meta-Analyses extension for Scoping Reviews (PRISMA-ScR) Checklist (Supplementary Table 1) [25].

### Protocol and registration

We followed the classic framework for scoping reviews by Arksey and O’Malley, as well as recent guidance to increase rigor and reporting of scoping reviews [25–27]. We developed our a priori protocol using the PRSIMA extension for Scoping Reviews as a guide [25]. Given the rapid timeframe of this review, we opted not to publish the protocol for this review, but it is available upon request.

### Eligibility criteria

We included studies that examined the validity of self-reported measures of cannabis use in pregnant women. More specifically, studies needed to compare estimates of self-reported prenatal cannabis use to a biochemical measure of cannabis use, including but not limited to hair, urine, and meconium sampling. We included studies from any geographical location if they were published in 2010 onward and written in English. We included only peer-reviewed articles regardless of study design, so long as they met other criteria, which included systematic reviews, with or without meta-analysis, and reviews of the literature. We excluded studies that did not include pregnant women only, were published before 2010, were published as conference abstracts or book chapters, or were not published in English. We also excluded studies reporting solely on the prevalence of prenatal cannabis use via self-report (e.g., national level surveillance data) or estimates of prevalence from biochemical samples, as comparison between self-report and a biochemical measure was not possible.

### Information sources

We systematically searched PubMed, PyschINFO, CINAHL, and Cochrane/CENTRAL from January 2010 to June 2021. We also included the first 200 results from Google Scholar, when sorted via relevance ranking. Given the shifting landscape of prenatal cannabis use, we limited our search from 2010 onward to identify contemporary measures of self-reported cannabis use, as opposed to dated measures that included cannabis in a category with other illicit substances (e.g., cocaine, heroin). We developed unique search strategies for each database, which we then piloted. After the initial pilot searches, we adapted the initial search terms to exclude those that did not yield relevant results, which included the following search terms: “survey”, “weed”, and “CBD”. We developed the final search strategy (Supplementary Table 2) using terms specific to our population (e.g., “pregnant”, “prenatal”, “pregnancy”) and topic (e.g., “cannabis” and “marijuana”) [28, 29], including terms to capture validation (“validity”, “evaluation”, “validation”, “agreement”) [25].

### Selection of sources of evidence

We used an online systematic review management software, Covidence, to streamline the review process (Covidence Systematic Review Software, Veritas Health Innovation, Melbourne, Australia). As the first step of our review process, we exported all search results from each database into EndNote (Clarivate Analytics, Philadelphia, USA). Next, we imported citations from EndNote (Clarivate Analytics, Philadelphia, USA) into Covidence. As part of this import process, Covidence automatically de-duplicated citations based on a match of the citation title, author, and date.

After search results were imported into Covidence, we performed a two-stage review process in which we screened references for inclusion based on eligibility criteria. To ensure reviewer agreement, two reviewers (KS and ED) piloted the screening process with 25 citations. Inter-rater agreement was high (95%) and thus formal screening began. Then, two members of the research team performed title and abstract screening independently. Upon completion of title and abstract screening, two reviewers screened remaining studies in their full-text, PDF form. Only articles meeting all inclusion criteria moved forward for data extraction. We resolved disagreements between reviewers at any stage using consensus and discussion. Lastly, we performed forward and backward citation searches for the final list of included studies.

#### Data extraction

Two reviewers independently performed data extraction for each included study using modifiable templates in Covidence. We abstracted the following datapoints from each study: study details (e.g., author, setting, dates, purpose, funding), population and sample size, study design and methods, details about measures used (both self-reported and biochemical), outcomes, limitations, recommendations (both for practice and research), and conclusions. Specific outcomes of interest included negative predictive value (NPV), positive predictive value (PPV), sensitivity, specificity, and percent agreement between maternal self-report and biochemical sampling. However, due to expected variation in reporting of outcomes, we included studies that reported other outcomes measuring the relation between maternal self-report and biochemical tests.

#### Synthesis of results

We performed a narrative synthesis, mapping existing evidence across key categories, including type of biochemical sample used for comparison of self-report and type of self-report used (e.g., health care provider screening, written screener, etc.). We also present data in tabular form by country and a separate table reporting recommendations for both future research and practice.

## Results

**Fig. 1:**
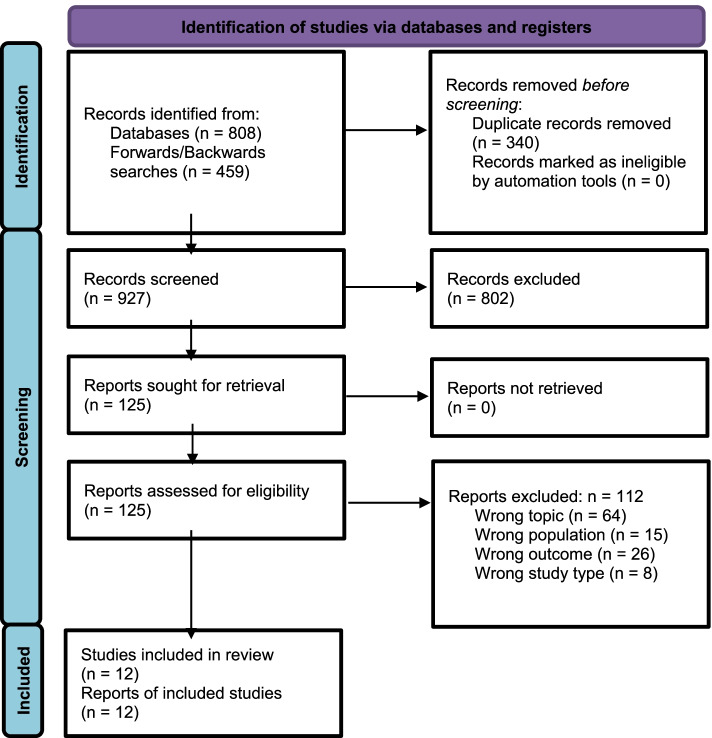
PRISMA Flow Diagram

After de-duplication, we screened a total of 927 unique articles, resulting in 12 articles included in this scoping review. We detail the study screening and selection process in accordance with PRISMA guidelines in Fig. 1.

### Study characteristics

We report key characteristics of included studies in Table 1. Of the 12 studies included, 7 were conducted in the US [17, 21, 23, 30–33] and 4 in other countries, including Brazil [34], the Netherlands [24], France [35], and South Africa [36]. The included systematic review contained studies conducted in an array of countries from across the globe [37]. Studies were heterogenous in overall study design, population, sample size, and measures used. Studies included pregnant women at different stages of pregnancy, ranging from the first prenatal visit to delivery. Sample sizes ranged from 83 [17] to 281,025 pregnant women [23].

**Table 1:** Key characteristics of included studies (*n* = 12)

Study ID	Setting	Aim(s)	Population (N)	Key Findings	Conclusions
**Primary Studies**					
***United States***					
Beatty et al. 2012 [30]	Large urban hospital in MI	To examine prenatal marijuana and tobacco use measured by self-report and compare marijuana use prevalence across self-report, urine drug assay, and hair sample testing	100 Women ages 18 years of age or older, English speaking, and had no postpartum administration of narcotic pain medication	Self-reported prevalence of any marijuana use was 11%, However, objectively defined marijuana use was more prevalent than self-reported tobacco use: 14% tested positive for marijuana by urinalysis, and 28% by hair analysis. A total of 14 participants were positive for past 3-week marijuana use: 10 by urine toxicology results only and 4 by both self-report and urine toxicology results.	Objective measures (urine and hair toxicology results) of recent and longer-term marijuana use revealed rates of marijuana use to be three times higher than was indicated by self-report. A broader public health response to address prenatal marijuana use is needed.
Chang et al. 2017 [31]	Five outpatient obstetrics and gynecology clinics in Pittsburg, PA	To examine audio-recorded 1st obstetric visits to assess rates of screening, disclosure, and use of prenatal marijuana and illicit drug use, comparing disclosure to urine screening	422 pregnant women 18 years of age or older, English speaking, and attending their first obstetric visit	*Screening****:*** OCPs asked about illicit drug use in 81%; 29% disclosed any current or past illicit drug use to their OCP. Among women who disclosed illicit drug use, 11% (*n* = 48) disclosed current marijuana use and another 7% (*n* = 30) disclosed past marijuana use.	Although marijuana is illegal in Pennsylvania, a high proportion of pregnant patients used marijuana, with many not disclosing use to their obstetric care providers. This highlights the limitations of perinatal illicit drug use studies that rely solely on self-report or medical record data.
Garg et al. 2016 [17]	University of New Mexico hospital-affiliated specialty prenatal clinic; Biomarkers in Pregnancy Study	To assess validity of self-reported drug use for major classes of illicit drugs and opioid-maintenance therapy among Hispanic and Native American pregnant women	83 pregnant women 18 years of age or older, fluent-English speaking, with a singleton pregnancy 35 weeks’ gestation or less	Prevalence of marijuana was (25.3%). Sensitivity of self-report for marijuana was 57.9%. Sensitivity of self-report for marijuana was higher among occasional users compared to regular users (42.9% vs. 38.5%). Specificity of self-report for all drug classes was high (≥ 90%), indicating that self-reported non-users were confirmed by negative urine drug screens in most cases.	Findings suggest that prenatal drug use is highly underreported, including among women who regularly participate in urine drug screenings. Though underreported, marijuana was more accurately reported than other drug classes. Future studies should be cautious about exclusive reliance on self-report.
Klawans et al. 2019 [32]	Urban, university-affiliated obstetric clinics in TX	To compare rates of prenatal marijuana and other illicit substance use via three identification and screening methods: self-report; urine drug screening ordered by clinician via routine practice; and universal urine drug screening	116 pregnant women presenting for first prenatal visit to the obstetric clinic between Aug and Dec 2015	Via self-report and universal urine drug screen results, 11.6% (*n* = 27) of the sample were current marijuana users. 80% of women with a marijuana-positive urine screen denied current use on survey; 75% of women who tested positive for marijuana via universal screening were not selected by clinicians for a urine screen. 90% of women who reported current marijuana use did not receive a clinician-ordered drug test.	Prenatal marijuana use was high, with limitations of patient self-report and selective, non-routine screening to identify prenatal substance use. Clinician-ordered tests increased identification by only 0.5% beyond self-report for marijuana. Effective, standardized, clinic-wide strategies are needed to support providers in identifying pregnant women who use substances to increase the frequency of education and intervention.
Metz et al. 2019 [21]	Two urban medical centers in Aurora and Denver, CO	To compare maternal marijuana use prevalence via self-reported to prevalence via umbilical cord sampling in a state with legalized marijuana. Secondary objective was to evaluate if reported frequency of use in the month prior to delivery was correlated with THC-COOH detection in the cord	116 women with a viable singleton pregnancy with 24 weeks or greater gestation who were admitted for delivery and delivered across 12 consecutive weekdays in Nov 2016	In the sample, 2.6% of patients reported marijuana use to health care providers; 14.7% reported past year use and 6.0% reported past-month use via survey. There was moderate agreement between 30-day use on the survey and umbilical cord homogenate above the limit of detection for THC-COOH (kappa 0.52, 95% CI 0.32–0.72). Agreement between disclosure to health care providers and self-reported use in the past year on the survey was fair (kappa 0.27, 95% CI 0.02–0.51). The agreement between medical record review and umbilical cord homogenate above the limit of detection for THC-COOH was slight (kappa 0.17, 95% CI 0.0–0.34).	Recent studies using umbilical cord testing have suggested an association between marijuana use and adverse outcomes including stillbirth and neonatal morbidity. Umbilical cord sampling results in higher estimates of prenatal marijuana use than self-report even in the setting of legalization. Thus, umbilical cord assays for THC-COOH demonstrate promise for quantifying use.
Yonkers et al. 2011 [33]	Integrated obstetrical/substance use treatment program, NR	To determine the relationship between self-report and urine toxicology tests of drug use over many time intervals prior to assessment of marijuana or cocaine use	168 pregnant women at least 16 years of age, English- or Spanish-speaking, not yet completing their 29th week of pregnancy, reported alcohol or illicit drug use other than opiates, during 28 days prior to screening or scored > 3 on the TWEAK	Mean reported frequency of past 30-day use for use of any hazardous substance other than nicotine was 5.5 days (SD = 8.3). Of those indicating past or current problems with marijuana, mean frequency of past 28-day use was 8.7 (SD = 9.3). Of the 69 women who tested positive for marijuana, 64% (*n* = 44) reported use within 1–10 days of the test, with 78% (*n* = 54) reporting some use within 28 days of the test. Of the 47 women who reported using marijuana 1–10 days before the test, 44 (94%) tested positive. Marijuana agreement between self-report and toxicology results for the prior month was k = 0.74 (95% CI = 0.63, 0.84).	Analysis of pregnant substance users found good agreement with one-month self-report of marijuana use and urine toxicology reports. Many women who screened positive reported use later than suggested by the toxicology screening test, particularly for cocaine. A question about use of marijuana or cocaine during the preceding month rather than the prior few days may be a better indicator of use. Positive past-month reports of drug use may indicate current drug use.
Young-Wolff et al. 2020 [23]	Two Kaiser Permanente Northern California (KPNC) medical centers, CA	To determine validity of self-reported prenatal cannabis use via comparison to positive urine toxicology testing, and predictors of nondisclosure using data from a large integrated healthcare delivery system with universal screening for prenatal cannabis use.	281,025 KPNC pregnant women who were screened for self-reported cannabis use during pregnancy between 2009 and 2017, and had a urine toxicology test for cannabis 2 weeks from the date they completed the self-reported screening questionnaire	Urine toxicology testing identified more instances of prenatal cannabis use than self-report (4.9% vs 2.5%). Older women, those of Hispanic race/ethnicity, and those with lower median neighborhood incomes were most likely to be misclassified as not using cannabis by self-reported screening. In our sample, self-reported screening correctly identified only 34% of those who had a positive urine toxicology test. About 2/3 of women tested positive for prenatal cannabis use by toxicology testing. We validated self-reported prenatal cannabis use using the urine toxicology test as the criterion standard; sensitivity of self-reported use was very low (33.9%) and the PPV of self-reported prenatal cannabis use was moderate (65.8%). Specificity of the urine toxicology test (99.1%) and NPV (96.7%) were excellent. Sensitivity of the toxicology test was higher (65.8%), with greater detection of self-reported daily (83.9%) and weekly (77.4%) than monthly or less use (54.1%).	Results from this study indicate that sensitivity of self-reported prenatal cannabis use during prenatal care is low and misclassification of use by self-report may vary with sociodemographic characteristics. Sensitivity of the urine toxicology test is higher, with greater detection of self-reported daily and weekly use versus monthly or less use. Given that many women chose not to disclose prenatal cannabis use in healthcare settings, it is important that clinicians educate all patients of reproductive age about the potential risks of prenatal cannabis use and advise prenatal patients to avoid using cannabis during pregnancy.
***Other Countries***					
Bessa et al. 2010 [34]	Labor and delivery unit of Mario Moraes Altenfelder Silva Maternity Hospital, São Paulo, Brazil	To check the validity of self-report of drug of pregnant adolescents, by comparing interview responses about cocaine and marijuana use with hair samples	1000 pregnant teenage inpatients ages 11 to 19	Hair analysis detected the use of cocaine and/or marijuana in the third trimester of the pregnancy in 6% (*n* = 60) of patients, with 4% (*n* = 40) using only marijuana, 1.5 (*n* = 17) used only cocaine and 0.3% (*n* = 3) used both drugs. None of the patients had reported the use of these substances in their interviews. 0% disclosure (0/957) reported cannabis use, but 43 tested positive.	Drug abuse during teenage pregnancy is a major health problem and the identification of infants born from these mothers should be done using sensitive methods of detection right after birth so that appropriate intervention can be performed.
El Marroun et al. 2011 [24]	Generation R study in Rotterdam, Netherlands	To verify self-reported information on prenatal drug use in urine	8880 women from prenatally enrolled population-based birth cohort within the Generation R study	Of the 3997 with urine samples available, 92 (2.3%) reported having used cannabis during pregnancy and 71 (1.8%) had positive urine screens. 35% of the 92 women with self-reported cannabis use also had a positive urine screen. Positive urines were frequent in women reporting cannabis use before pregnancy only (7.6%) and in women with missing information (2.6%). Sensitivity and specificity of urinalysis compared to self-report were 0.46 and 0.98. Sensitivity and specificity of self-report compared to urinalysis were 0.36 and 0.99. Yule’s Y amounted to 0.77, indicating substantial agreement between the two measures. Compared to women that did not report use, paternal cannabis use was more common in women disclosing use (78.9 vs. 8.5%; *p* < 0.001). Paternal cannabis use was also more common when maternal urine samples were positive (71.7 vs. 9.2%; *p* < 0.001)	These findings indicate that both approaches perform very well in the identification of non-cannabis users, but that both measures seem to identify partially different subpopulations of cannabis users during pregnancy. In conclusion, researchers and clinicians should acknowledge that pregnant women may underreport current cannabis use, a situation that seems most prevalent in women admitting past cannabis use and in women refusing to provide information on prenatal cannabis use. Findings illustrate the difficulties in obtaining valid information on prenatal cannabis use; self-report seems to be an acceptable single method to determine cannabis use during pregnancy in epidemiological studies.
Lamy et al. 2017 [35]	Maternity hospitals in Rouen, Normandy, France	To compare self-reported prevalence of alcohol, tobacco and/or cannabis use during the third trimester of pregnancy with results of meconium testing of their metabolites in newborns	724 pregnant women aged 18 or over, living in our catchment area that delivered a child in one of these maternity hospitals	Cannabis use prevalence was low via self-report (0.8%, *n* = 6); and meconium sampling (1.1%, *n* = 7). We found a low level of concordance (Kappa = 0.30) between cannabinoid metabolites in meconium samples and self-reports of cannabis use during the 3rd trimester. Only 2/7 positive meconium samples were concordant with self-reports (1–2 joints/day during the 3rd trimester). In all 7 cases, cotinine was also positive. In 3 women, including 2 women reporting daily use in 3rd trimester reporting prenatal cannabis use, cannabinoid metabolites were negative in meconium. One pair of dizygotic twins had positive cotinine, EtG and THC-COOH meconium samples, with EtG and THC-COOH concentrations 6 and 2 times higher in the female twin compared to the male twin, respectively.	Maternal psychoactive substance use is an ongoing concern; detecting prenatal use is a crucial component of early diagnosis of fetal alcohol syndrome and neonatal care. There was almost no concordance between maternal self-reports of cannabis use and THC-COOH quantitative measures. Assessment of prenatal cannabis exposure, using meconium testing needs to be improved.
Williams et al. 2020 [36]	Community-based clinics called midwife obstetric units in Greater Cape Town (Metropole), South Africa	To examine agreement among simple dichotomous self-report, validated screening results, and biochemical screening results of prenatal alcohol and other drug use	684 pregnant women 16 years or older, presenting for prenatal care	The weighted sensitivity for ASSIST self-report of cannabis use compared to urine screening biomarkers was 51.4% (95% CI: 27.8–74.9), and the specificity was 98.4% (95% CI: 97.7–99.2) with the PPV being 37.6% and the NPV being 99.1%.	Self-reported prevalence of illicit drug use was underreported. Combined use of urine screenings and self-report can be recommended especially for identifying underreported substances to accurately detect AOD use in pregnancy, to enable identification and referral to intervention(s) can occur.
**Systematic Reviews**					
Chiandetti et al. 2017 [37]	Global: US, Spain, Canada, Sweden, Italy, Denmark, Uruguay	To compare reported rates of prenatal alcohol and drugs of abuse exposure with biomarkers of exposure by a comprehensive review of available literature	Studies published in English between 1992 and 2015. Inclusion criteria was “diagnosis/identification/detection of prenatal exposure to drugs of abuse or alcohol”.	Studies agreed that either meconium or hair analysis proved more sensitive than maternal interview for drugs of abuse. Garcia-Serra et al. found more sensitivity in hair analysis than maternal meconium to detect cannabis. The percentage of women who admitted to using THC was 2.9%. Positive results in biomarkers were up to 4% for THC, whilst Lendorio et al. found up to 12.4% positives for THC.	We propose using biomarkers as the main screening tool in patients in environments with high prevalence of AOD of abuse, along with questionnaires. Studies with biomarkers may not be available in all services but should be considered in cases with suspected use even if denied in questionnaires.

*Abbreviations*: *AOD* Alcohol and other drug, *NPV* Negative predictive value, *NR* Not reported, *OCP* Obstetric care providers, *PPV* Positive predictive value, *THC* Tetrahydrocannabinol

### Validity outcomes

#### Urine

Most included studies (*n* = 8) compared maternal self-reported prenatal cannabis use prevalence to urine [17, 23, 24, 30–33, 36]. Overall agreement between maternal self-report and urine was poor to moderate, ranging from 34% [23] to 64% [33]. Young-Wolff et al. (2020) reported the lowest agreement, with self-report screening identifying only 34% of those testing positive via urine [23]. Similarly, El Marroun et al. (2011) found poor agreement between self-report and urine, with only 35% of the 92 women reporting cannabis use had positive urine screens [24]. Similarly, Chang et al. (2017) found that only 36% of women with a positive urine screen disclosed use to a health care provider [31]. Garg et al. (2016) and Yonkers et al. (2011) found the highest level of agreement between self-report and urine toxicology, with 60 and 64% agreement, respectively [17, 33].

#### Hair

Hair analysis was used in 2 of the included studies [30, 34], with both studies reporting poor agreement of self-reported prenatal cannabis use. One study conducted in Brazil found no agreement with hair samples due to a 0% disclosure rate for cannabis [34]. Another study conducted in the US found overall prevalence of cannabis use via hair sampling was 28% (compared to 11% via self-report only); 6 participants who reported cannabis use had a negative hair sample [30].

#### Umbilical cord

A single study compared maternal self-reported cannabis use to umbilical cord homogenate assays, comparing biochemical results to both survey and medical record review [21]. Metz et al. (2019) found moderate agreement between 30-day use via survey and umbilical cord homogenate (kappa = 0.52) and slight agreeance between medical record review and umbilical cord homogenate (kappa = 0.17) [21].

#### Meconium

Lamy et al. (2017) was the only included study that examined maternal self-report prevalence to meconium samples [35]. In this study, overall concordance between self-report of 3rd trimester cannabis use and cannabinoid metabolites in meconium samples was low (Kappa = 0.30). In fact, 2 women who reported daily use during the 3rd trimester of pregnancy were negative for meconium cannabinoid metabolites.

### Type of self-report

#### Health care provider screening

Two studies relied on health care provider verbal screening for self-reported cannabis use. Chang et al. (2017) recorded first obstetric visits for assessment of disclosure of cannabis use via health care provider verbal screening and found that 74% of patients who tested positive for cannabis did not disclose use [31]. Another study assessed disclosure of cannabis use to a health care provider and found fair agreement between self-report and umbilical cord homogenate (kappa 0.27, 95% CI 0.02–0.51) [21].

#### Interview (structured or semi-structured)

A total of 5 studies utilized structured or semi-structured interviews to assess self-reported cannabis use [17, 33–36] and found poor to moderate agreement with estimates via biochemical sampling. One study assessed self-report using a semi-structured interview conducted by trained midwifery students via the French version of the 5th Edition of the Addiction Severity Index and found a low level of agreement between self-report and meconium sampling (Kappa = 0.30) [35]. Another study by Bessa et al. (2010) found that of pregnant adolescents testing positive for cannabis, none disclosed use [34]. Williams et al. (2020) utilized the Alcohol, Smoking and Substance Involvement Screening Test (ASSIST) for assessment of self-reported cannabis use and found the positive predictive value to be only 37.6%. Garg et al. (2016) found that nearly 60% of participants disclosed cannabis use [17]. Importantly, in this study, trained researchers with no clinic affiliation interviewed patients at a clinic serving patients with a current or past history of substance use [17]. Yonkers et al. (2011) had researchers perform intake assessments in a sample of pregnant women who reported substance use and found that the agreement between self-report and urine toxicology was moderate (Kappa = 0.74).

#### Self-administered questionnaires

Three studies used a written survey to assess self-reported cannabis use [21, 23, 32]. Young-Wolff et al. (2020) found that self-reported screening correctly identified only 34% of those who had a positive urine toxicology test [23]. Klawans gave participants a written survey that assessed cannabis use and found that although 27 women (11.6%) tested positive for cannabis via universal screening, only 10 women (4.6%) reported current cannabis use [32]. Beatty et al. (2012) used audio-enhanced computer-assisted self-interview (ACASI) technology to screen for self-reported use, in which biological measures of cannabis use (both hair and urine) revealed actual prevalence of use to be 3 times higher than self-report [30].

### Research and practice recommendations

Included studies had numerous recommendations for both future research and practice on this topic (Table 2). Several studies called for more research on the validity of maternal self-reported prenatal cannabis use specifically in larger samples that are more diverse to improve generalizability of findings [17, 23, 33]. Most recommendations were focused on integrating study findings into clinical practice. The most cited recommendation for clinical practice was utilization of both self-report and biochemical estimates of use to improve overall identification of cannabis use [17, 23, 24, 36, 37]. A common area of future research recommendations included identify factors associated with perinatal illicit drug disclosure and how these factors impact sensitivity and accuracy of self-report [17, 31, 38]. Several studies also recommended further research on maternal self-report using more representative samples [17, 23, 33].

**Table 2:** Research and practice recommendations of included studies

Research	Practice
Assess effectiveness of different screening methods perinatal illicit drug use prevention [31]	Health care providers should consider the environment which the reporting occurs as it can influence the disclosure of sensitive information [34]
Identify factors associated with perinatal illicit drug disclosure and how these factors impact sensitivity and accuracy of self-report [17, 31, 38]	As neither method is perfect, a combination of self-report and biomarkers is recommended to best detect prenatal cannabis use [17, 23, 24, 36, 37]
Examine screening and testing methods that promote conversation instead of punitive recourse [31]	Supporting clinicians via clinic-wide, standard substance use screening policies is essential to improving overall health care [32]
Examination of validity of maternal self-report in larger, more representative cohorts of pregnant women [17, 23, 33]	Health care providers should educate pregnant women about potential risks and common misconceptions of cannabis use and advise cessation [23, 30]
Examination of how biological sampling may inform the relationship between cannabis use and perinatal outcomes [21]	Health care providers need to be alert and use clinical judgement when determining a patient’s drug use and need for an intervention [33]
Evaluate health care providers’ counsel of patients using illicit drugs, including how provider responses vary depending on the drug, and how responses affect perinatal substance use [31]	Current strategies need improvement; effective and standardized methods to identify perinatal cannabis use and exposure are needed to inform interventions [31, 32, 35]
Further robust studies on the effects of prenatal marijuana exposure [30]	Earlier and more sensitive methods of detecting prenatal cannabis is critical in implementing interventions and prevention adverse health effects [34, 37]
	Clinician-ordered urine screenings have the potential for low sensitivity and specificity, likely indicating a degree of insufficiency in accurate identification of women who use substances [32]
	Cannabis policies within a given region need to be considered when screening patients, as the negative consequences related to illegal use during pregnancy may impact disclosure, as well as treatment and support services [31]

## Discussion

In this scoping review, we identified and synthesized contemporary evidence on the validity of maternal self-reported cannabis use during pregnancy. We found 12 studies that examined the validity of self-reported prenatal cannabis use in comparison to a biochemical estimate. Most studies were conducted in the US and conducted in either a hospital or clinical setting. The most commonly used biochemical measure used was urine testing, which leaves substantial gaps relating to the evidence on validity of self-report compared to other biochemical measures, such as hair, meconium, or umbilical cord sampling. Given the potential adverse maternal and child health effects of prenatal cannabis exposure, our findings necessitate additional research examining validity of self-reported prenatal cannabis use.

Accurate identification of women who use cannabis during pregnancy is imperative for prenatal care providers so that discussions about use and referral to treatment, if necessary, can occur. Undoubtedly, this cannot occur without utilization of valid measures of prenatal cannabis use. However, in our review, we found that self-report of prenatal cannabis use was largely unreliable. Consistent with prior studies, we found that biometric estimates found higher prevalence of prenatal cannabis use compared to self-report. Although biometric estimates of prenatal cannabis use are more resource and time-intensive compared to self-report measures [39], several included studies recommended that a combination of self-report and biochemical screening should be employed by clinicians to improve accuracy of identifying women who use or are exposed to cannabis during pregnancy [17, 23, 24, 36, 37]. Indeed, evidence supports that indirect cannabis exposure can lead to positive biochemical samples for metabolites of the drug [40].

Prior research has shown that ACASI approaches have been associated with increased disclosure of substance use [41, 42]. However, we did not find this to be true; one included study using ACASI found that biochemical estimates revealed nearly three times the amount of cannabis users as self-report [30]. Interestingly, Yonkers et al. (2011), who used interviews to assess self-reported cannabis use, had the highest agreement between self-report and urine toxicology (kappa = 0.74), which perhaps was due to their population of pregnant women from an integrated obstetrical/substance use treatment program [33]. The second highest congruence between self-report and biochemical estimates were reported in another study utilizing interviews for self-report in a clinic serving patients with a current or history of substance use and found approximately 60% disclosed use. The high level of agreement in Garg et al. (2016) is likely attributable to the absence of punitive consequences for participants in their study and perhaps the population as well [17]. Agreement between self-reported cannabis use and biochemical estimates were lowest in studies utilizing health care provider screening [21, 31]. Importantly, in several studies, women knew they would be subsequently tested for cannabis after self-reporting use. In turn, these studies may report agreement levels that are higher than typical agreement.

We found several evidence gaps, which future research should work to address. As there was only one review that examined maternal self-report to meconium samples [35], we found insufficient evidence to comprehensively examine the validity of self-report in comparison to this type of biochemical measure. We found that self-report was more reliable in populations with a current or prior history of drug use and when assessed via interviews compared to health care provider screenings and self-administered surveys. As there are many factors influencing the agreement between self-report and biochemical estimates of cannabis use, such as social norms, fear of punitive action, and metabolite detection methods, future research should aim to better understand these factors. Beyond standardized clinic protocols for screenings and discussions of prenatal cannabis use, another important point of consideration for future studies is to examine the extent to which setting, population, and health care provider characteristics are associated with the validity of self-reported prenatal cannabis use, as we did not find a single study examining this relation.

To meet the aims of this study, we determined a scoping review, as opposed to a systematic review, was the best approach for several reasons. First, scoping reviews are used to determine the breadth and depth of existing evidence on a topic through systematic identification and mapping of available evidence [26, 27]. Secondly, scoping reviews are ideal to identify any knowledge gaps as well as to pinpoint specific research questions that could be answered via a more precise systematic review [27, 43]. Accordingly, an aim of this scoping review was to determine if there was enough evidence for, and to specify the research questions of, a systematic review on this topic. Indeed, our review suggests enough evidence for a systematic review. A systematic review on this topic would be able to provide a meticulous summary of available primary research that clinicians can use to develop prenatal cannabis use screening guidelines and policies. Until such a review is undertaken, prenatal health care providers are left to navigate the inherent complexities of shifting cannabis policies and increases in prenatal cannabis use in murky waters. Specifically, our findings support a systematic review that aims to answer the following research questions:

1. What is the validity and reliability of self-reported cannabis use during pregnancy?;
2. How is the accuracy of biochemical estimates of cannabis use impacted by cannabinoid pharmacokinetics variability and metabolite detection methods (e.g., point of care testing, mass spectrometry)?;
3. How does accuracy of self-reported cannabis use during pregnancy vary across environmental factors (e.g., cannabis legalization, setting, health care provider traits)?;
4. What is the extent to which validity of self-reported cannabis use varies as a function of time between collection of self-reported and biochemical samples?;
5. What potential harms or adverse outcomes exist for screening of prenatal cannabis use (both self-report and biochemical estimates)? How do these harms or adverse outcomes vary across cannabis policies?

### Limitations

There are a few limitations of this scoping review. First, we excluded studies not published in English, which likely resulted in failure to identify all potentially relevant studies. Second, we utilized date restrictions to capture recent studies with contemporary relevancy (e.g., delineate cannabis from other illicit substances, use non-stigmatizing language). However, by using date restrictions in the search, we may have missed in-press or recently published articles yet to be indexed. Among included studies, there was inconsistency in the reported measure for agreement, with some studies reporting agreement in the form of Cohen’s kappa and others reporting sensitivity or specificity. A future systematic review can aim to address this limitation by calculating a consistent measure of agreement across studies for comparison. Another important piece to consider when comparing biochemical estimates in comparison to self-report is the window of time between collection of the two measures. This was beyond the scope of this review but is an important question to answer in a systematic review with possible meta-analysis. Lastly, the small number of studies that used meconium or umbilical cord sampling precluded a proper synthesis of studies for those measures.

## Conclusion

We conducted a scoping review to identify and map available evidence on the validity of self-reported prenatal cannabis use. We found validity of self-report was poor in comparison to biochemical estimates. Further research is urgently needed to understand and examine factors associated with the validity of self-reported prenatal cannabis use, as well as to develop valid measures of self-reported use. Additionally, a systematic review is urgently needed to guide clinical practice and policy. Until this necessary research can be conducted, clinicians should use the recommendations of prior studies as outlined above.

## Supplementary Information

**Additional file 1.**

**Additional file 2.**

## Data Availability

The protocol for this review can be accessed by emailing the corresponding author. All articles included in this review can be accessed online.

## Abbreviations

ACASI: Audio-enhanced Computer-assisted Self-interview
ASSIST: Alcohol, Smoking and Substance Involvement Screening Test
CENTRAL: The Cochrane Central Register of Controlled
NPV: Negative predictive value
PPV: Positive predictive value
PRISMA: Preferred Reporting Items for Systematic Reviews and Meta-Analyses
US: United States
