# Supplementary material for: Validity of self-report measures of cannabis use compared to biological samples among women of reproductive age: a scoping review
Source: BMC Pregnancy Childbirth. 2022 Apr 21;22:344. doi: 10.1186/s12884-022-04677-0 (PMC9027056; doi:10.1186/s12884-022-04677-0)
Supplement: Supplementary file 2 — Additional file 2. [file 12884_2022_4677_MOESM2_ESM.docx]

**Additional File 1: Search Strategy**

Lines 1 to 7 (in black) detail the final search strategy used for this review. The remaining searches in **blue**, detail the full pilot search strategy. The search strategy carried out using a mixture of controlled vocabulary and key words.

| 1 | TI (valid* or evaluation or agreement) OR AB (valid* or evaluation or agreement) |
| --- | --- |
| 2 | TI (prenatal or pregnan*)OR AB (prenatal or pregnan*) |
| 3 | TI (marijuana OR cannabis) OR AB (marijuana OR cannabis) |
| 4 | 1 AND 2 |
| 5 | 3 AND 4 |
| 6 | TI (perinatal) AND AB (perinatal) |
| 7 | 5 AND 6 |
| 8 | TI (weed or pot or CBD) OR AB (weed or pot or CBD) |
| 9 | 7 AND 8 |
| 10 | TI (screener) |
| 11 | 9 AND 10 |
| 12 | 2 AND 12 |
| 13 | TI (weed or pot or CBD) OR AB (weed or pot or CBD) |
